# The Carboxyl-Terminus of TRANSPARENT TESTA GLABRA1 Is Critical for Its Functions in Arabidopsis

**DOI:** 10.3390/ijms221810039

**Published:** 2021-09-17

**Authors:** Yating Wang, Hainan Tian, Wei Wang, Xutong Wang, Kaijie Zheng, Saddam Hussain, Rao Lin, Tianya Wang, Shucai Wang

**Affiliations:** 1 Laboratory of Plant Molecular Genetics & Crop Gene Editing, School of Life Sciences, Linyi University, Linyi 276000, China; wangyt814@nenu.edu.cn (Y.W.); wangw716@nenu.edu.cn (W.W.); wangxt357@nenu.edu.cn (X.W.); 2 Key Laboratory of Molecular Epigenetics of MOE, Northeast Normal University, Changchun 130024, China; tianhainan2012@gmail.com (H.T.); zhengkaijie@iga.ac.cn (K.Z.); hase705@nenu.edu.cn (S.H.); linr944@nenu.edu.cn (R.L.); wangty309@nenu.edu.cn (T.W.); 3 Key Laboratory of Soybean Molecular Design Breeding, Northeast Institute of Geography and Agroecology, Chinese Academy of Sciences, Changchun 130102, China

**Keywords:** TTG1, trichome formation, root hair formation, secondary metabolism, transcription factor, CRISPR/Cas9 gene editing, Arabidopsis

## Abstract

The Arabidopsis WD40 repeat protein TRANSPARENT TESTA GLABRA1 (TTG1) regulates cell fate determination, including trichome initiation and root hair formation, as well as secondary metabolism such as flavonoid biosynthesis and seed coat mucilage production. TTG1 regulates different processes via regulating the expression of its downstream target genes by forming MYB-bHLH-WD40 (MBW) activator complexes with different R2R3 MYB and bHLH transcription factors. Here, we report the identification of the carboxyl (C)-terminus as a critical domain for TTG1′s functions in Arabidopsis. We found that the *ttg1Δ15aa* mutant shows pleiotropic phenotypes identical to a *TTG1* loss-of-function mutant. Gene sequencing indicates that a single nucleotide substitution in *TTG1* led to a premature stop at the W327 residue, leading to the production of a truncated TTG1 protein with a deletion of the last 15 C-terminal amino acids. The expression of *TTG1* under the control of its native promoter fully restored the *ttg1Δ15aa* mutant phenotypes. Consistent with these observations, the expression levels of TTG1 downstream genes such as *GLABRA2* (*GL2)* and *CAPRICE* (*CPC*) were reduced in the *ttg1Δ15aa* mutant. Assays in Arabidopsis protoplast show that TTG1Δ15aa failed to interact with the bHLH transcription factor GL3, and the deletion of the last 3 C-terminal amino acids or the 339L amino acid alone fully abolished the interaction of TTG1 with GL3. Furthermore, the expression of *TTG1Δ3aa* under the control of *TTG1* native promoter failed to restore the *ttg1Δ15aa* mutant phenotypes. Taken together, our results suggest that the C-terminal domain of TTG1 is required for its proper function in Arabidopsis.

## 1. Introduction

TRANSPARENT TESTA GLABRA1 (TTG1) is a WD40 repeat protein with four WD40 motifs [1]. It is well known that TTG1 regulates cell fate determination including trichome and root hair formation and secondary metabolism such as flavonoid biosynthesis and seed coat mucilage production [2]. Arabidopsis loss-of-function mutants of *TTG1* show a pleiotropic phenotype including glabrous leaves, increased root hairs, transparent testa seed coat, reduced anthocyanin accumulation, and seed coat mucilage production [1,3,4,5,6]. In addition, studies in recent years have also shown that TTG1 is involved in the regulation of other biological processes, such as the accumulation of fatty acids and proteins during the seed maturation [7,8], flowering time [9], as well as plant responses to biotic and abiotic stresses [10,11,12].

The presence of WD40 motifs is the only common feature of the WD40 proteins, and the WD40 motifs are able to provide a platform for the interactions of WD40 proteins and other proteins [13,14,15]. Indeed, the available evidence suggests that, at least in the terms of regulating cell fate determination and secondary metabolism, TTG1 is able to interact with different basic helix-loop-helix (bHLH) transcription factors and with different R2R3 MYB transcription factors to form different MYB-bHLH-WD40 (MBW) activator complexes, thereby regulating the expression of its downstream target genes involved in cell fate determination or secondary metabolism [16,17,18,19,20].

In regulating trichome formation, MBW activator complexes formed by TTG1, the bHLH transcription factor GLABRA3 (GL3) or ENHANCER OF GLABRA3 (EGL3), and the R2R3 MYB transcription factor GLABRA1 (GL1) activate the expression of trichome regulator genes, including the homeodomain gene *GLABRA2* (*GL2,)* and some of the *R3 MYB* genes, including *TRYPTICHON* (*TRY*), *CAPRICE* (*CPC*), *ENHANCER OF TRY AND CPC1* (*ETC1*), and *ETC3* [17,18,19,21,22]. In regulating root hair formation, MBW activator complexes formed by TTG1, the bHLH transcription factor GL3 or EGL3, and the R2R3 MYB transcription factor WERWOLF (WER) activate the expression of root hair regulator gene *GL2* [19,23,24]. In regulating anthocyanin and proanthocyanidin biosynthesis, MBW activator complexes formed by TTG1, the bHLH transcription factors TRANSPARENT TESTA 8 (TT8), GL3 or EGL3, and the R2R3 MYB transcription factors PRODUCTION OF ANTHOCYANIN PIGMENT 1 (PAP1), PAP2, MYB113, MYB114, or TT2 activate the expression of the late biosynthesis genes in the flavonoid biosynthesis pathway, including *DIHYDROFLAVONOL 4-REDUCTASE* (*DFR*), *ANTHOCYANIDIN SYNTHASE* (*ANS*) and *UDP-GLUCOSE:FLAVONOID 3-O-GLUCOSYLTRANSFERASE* (*UF3GT*), *BANYULS* (*BAN*), *TT19,* and *TT12* [16,18,25,26,27,28,29,30,31]. However, in regulating seed coat mucilage production, the MBW activator complexes formed by TTG1, the bHLH transcription factor TT8 or EGL3, and the R2R3 MYB transcription factor MYB5 or TT2 activate the expression of mucilage biosynthesis genes *TTG2* and *GL2* [16,32,33,34,35,36].

Some experiments suggest that the formation of the MBW activator complexes may not be required for the activation of TTG1 downstream genes. For example, it has also been shown that the interaction of a R2R3 MYB and a bHLH transcription factor are required and sufficient to activate the expression of *GL2* and some *R3 MYB* genes [37], and instead to form MBW activator complexes, TTG1 may compete with GL1 for binding GL3, therefore to form GL1-GL3 or GL3-TTG1 dimers [38]. Yet experiments in both yeast and plant cells show that TTG1 is able to interact with bHLH transcription factors [39,40], and the presence of TTG1 enhanced the interactions between R2R3 MYB and bHLH transcription factors [25,39].

The domains required for the interaction of R2R3 MYB and bHLH transcription factors have been well studied. It has been shown that R2R3 MYB proteins interact with bHLH transcription factors via their R3 domains, and the [D/E]L × 2[R/K] × 3L × 6L × 3R in R3 domain is the amino acid signature required for these interactions [39,40,41]. On the other hands, the N-terminal domain of bHLH transcription factors is required for the interaction of bHLH transcription factors with R2R3 MYB proteins [40]. However, the domain required for TTG1 to interact with bHLH transcription factor remains largely unstudied. So far, more than 20 mutants of *TTG1* gene have been identified. Some loss-of-function mutants are caused by amino acid substitution, and the premature stop of TTG1 [2] suggests that the interaction of TTG1 and bHLH proteins may be affected in those mutants. However, it has only been shown that TTG1 with a deletion of the 25 carboxyl (C)-terminal amino acids, a truncated TTG1 protein corresponding to that in the *ttg1-1* mutant [1], failed to interact with GL3 [39], indicating that the C-terminal domain is required for the interaction of TTG1 with bHLH transcription factors.

We report here the identification of *ttg1Δ15aa*, a new mutant of *TTG1*, and the characterization of C-terminus in the interaction of TTG1 with the bHLH transcription GL3. We found that *ttg1Δ15aa* is a *TTG1* loss-of-function mutant, and assays in transfected protoplasts show that TTG1Δ15aa failed to interact with GL3. Further assays show that the deletion of the last three C-terminal amino acids or the 339L amino acid alone fully abolished the interaction of TTG1 with GL3, and the expression of *TTG1Δ3aa* under the control of *TTG1* native promoter failed to restore the *ttg1Δ15aa* mutant phenotypes. There results suggest that the last few C-terminal amino acids are critical for TTG1′s functions in Arabidopsis.

## 2. Results

### 2.1. Phenotypes in the ttg1δ15aa Mutant Are Identical to That in A TTG1 Loss-of-Function Mutant

**Figure 1:**
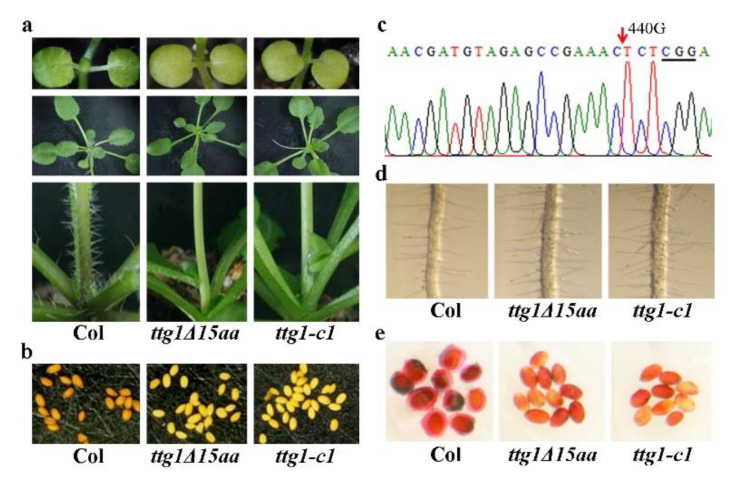
Phenotypes of the *ttg1Δ15aa* mutant and a *TTG1* gene-edited mutant. (**a**) Trichome formation on leaves of 10-day-old seedlings (up panel), 4-week-old plants (middle panel), and stems of 5-week-old plants (lower panel) of the Col wild type, the *ttg1Δ15aa*, and the *ttg1-c1* mutants. Seeds of the Col wild type, the *ttg1Δ15aa*, and the *ttg1-c1* mutants were germinated and grown in soil pots. Pictures were taken directly or under a Motic K microscope by using an EOS 1100D digital camera at indicated stages. (**b**) Seed color of the Col wild type, the *ttg1Δ15aa* and the *ttg1-c1* mutants. Pictures were taken under a Motic K microscope by using an EOS 1100D digital camera. (**c**) Editing status of TTG1 in the *ttg1-c1* mutant. The arrow indicates the 440 G deletion in the *ttg1-c1* mutant. Underline indicates the PAM site. The mutant was obtained by transforming the Col wild type plants with the *pHDE-TTG1* CRISPR/Cas9 construct. Editing status of *TTG1* was examined in T1 plants, and Cas9-free homozygous mutant plants were obtained in T2 generation. (**d**) Root hair formation in the Col wild type, the *ttg1Δ15aa*, and the *ttg1-c1* mutants. Seeds of Col wild type, the *ttg1Δ15aa*, and the *ttg1-c1* mutants were germinated and grown on half MS plates vertically. Pictures were taken from 7-day-old seedlings under a Motic K microscope by using an EOS 1100D digital camera. (**e**) Mucilage production in the Col wild type, the *ttg1Δ15aa*, and the *ttg1-c1* mutants. Seeds were stained with 0.01% (*w/v*) Ruthenium red for 2 h, and pictures were taken under a Motic K microscope by using an EOS 1100D digital camera.

From an *ethyl methanesulfonate* (EMS) mutant pool in the Col ecotype background, we identified a mutant with glabrous phenotype (Figure 1a). The mutant was named *ttg1Δ15aa*, as a single nucleotide substation in *TTG1* led to production of a truncated TTG1 protein with a deletion of the last 15 C-terminal amino acids (see Section 2.2 for details). In addition to glabrous leaves and stems, the *ttg1Δ15aa* mutant also has defects in anthocyanin accumulation (Figure 1a), as well as seed proanthocyanidin production (Figure 1b) and typical phenotypes observed in the *TTG1* loss-of-function mutants [1,3,4,5,16].

To examine if *ttg1Δ15aa* mutant is a *TTG1* loss-of-function mutant, we generated CRISPR/Cas9 gene edited mutant in the Col wild type background and compare their phenotypes side by side. In the *ttg1-c1* mutant, a single G nucleotide deletion occurred at the position of 440 relative to the start codon of *TTG1* (Figure 1c). As shown in Figure 1a,b, the *ttg1Δ15aa* mutant is identical to the *ttg1-c1* mutant in terms of trichome formation, anthocyanin accumulation, and seed proanthocyanidin production. Both of the *ttg1Δ15aa* and the *ttg1-c1* mutants also produced more root hairs (Figure 1d) and have defects on seed coat mucilage biosynthesis (Figure 1e).

### 2.2. A Single Nucleotide Substitution Led to the Production of A Truncated TTG1 Protein in the ttg1δ15aa Mutant

**Figure 2:**
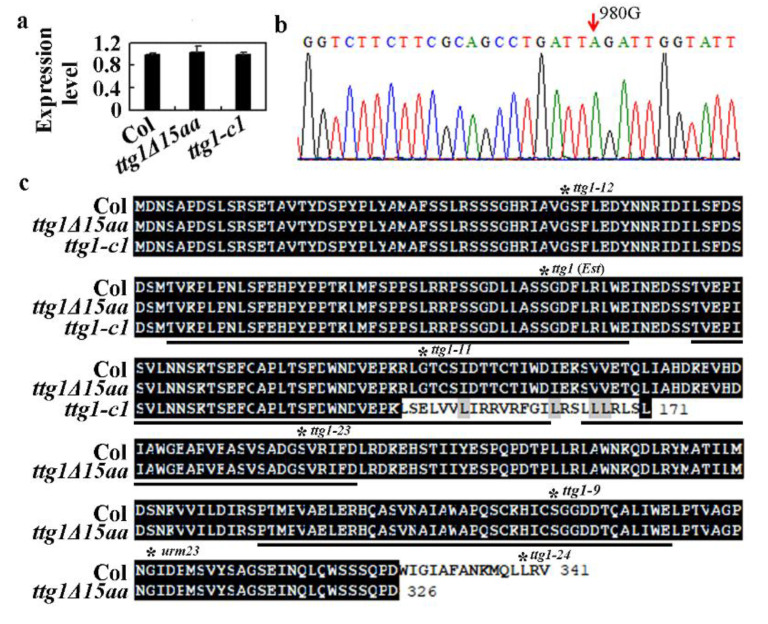
A single nucleotide substation in *TTG1* led to premature stop in the *ttg1Δ15aa* mutant. (**a**) Expression of *TTG1* in the Col wild type, the *ttg1Δ15aa*, and the *ttg1-c1* mutants. RNA was isolated from 10-day-old seedlings, and qRT-PCR was used to examine the expression of *TTG1*. The expression of *ACT2* was used as an inner control, and the expression level of *TTG1* in the Col wild type was set as 1. Data represent the mean ± SD of three replicates. (**b**) Sequence of *TTG1* in the *ttg1Δ15aa* mutant. The arrow indicates the 980G-A single-base substitution. (**c**) Amino acid alignment of TTG1 in the Col wild type, the *ttg1Δ15aa*, and the *ttg1-c1* mutants. ORFs of *TTG1* in the *ttg1Δ15aa* and the *ttg1-c1* mutants were identified by using DNAMAN, and the predicted full-length amino acid sequences were used for alignment with the full-length amino acid sequence of TTG1 in the Col wild type. The numbers at the C-terminal indicate the numbers of total amino acids of TTG1 in the Col wild type, the *ttg1Δ15aa*, and the *ttg1-c1* mutants. Solid underlines indicate the WD40 domains. Stars indicate the amino acids that were substituted in the corresponding mutants as summarized previously [2].

The above results suggest that *ttg1Δ15aa* is likely a loss-of-function mutant of *TTG1*. To examine if altered expression level of *TTG1* may be responsible for the phenotype’s observation in the *ttg1Δ15aa* mutant, we examined the expression of *TTG1* by using qRT-PCR. As show in Figure 2a, the expression level of *TTG1* in seedlings of the *ttg1Δ15aa* mutant is largely indistinguishable from that in the Col wild type seedlings. Consistent with the fact of *ttg1-c1* as a CRISPR/Cas9-edited mutant with only a single nucleotide deletion in *TTG1*, the expression level of *TTG1* in the *ttg1-c1* mutant is also similar to that in the Col wild type seedlings (Figure 2a).

Having shown that the phenotypes in the *ttg1Δ15aa* mutant are not caused by a reduced expression of *TTG1* (Figure 2a), we then amplified and sequenced the genome sequence of *TTG1* in the *ttg1Δ15aa* mutant. We found that the 980 G nucleotide was substituted by a nucleotide A (Figure 2b), therefore creating a TAG stop codon at the W327 residue, resulting in the production of truncated TTG1 protein with a deletion of the 15 C-terminal amino acids (Figure 2c). We thus named the mutant *ttg1Δ15aa*. As shown in Figure 2c, the deletion of the 15 amino acids did not affect any of the 4 WD40 repeats in TTG1 protein, whereas the nucleotide deletion in the *ttg1-c1* mutant led to a few amino acid substitutions in the second WD40 repeat, and a premature stop occurred after the 171 amino acid residue. For comparison, mutations in all the known mutants with an amino acid substitution in TTG1, as summarized recently [2], were indicated in Figure 2c.

### 2.3. Expression of TTG1 under the Control of its Native Promoter Restored the Ttg1δ15aa Mutant Phenotypes

**Figure 3:**
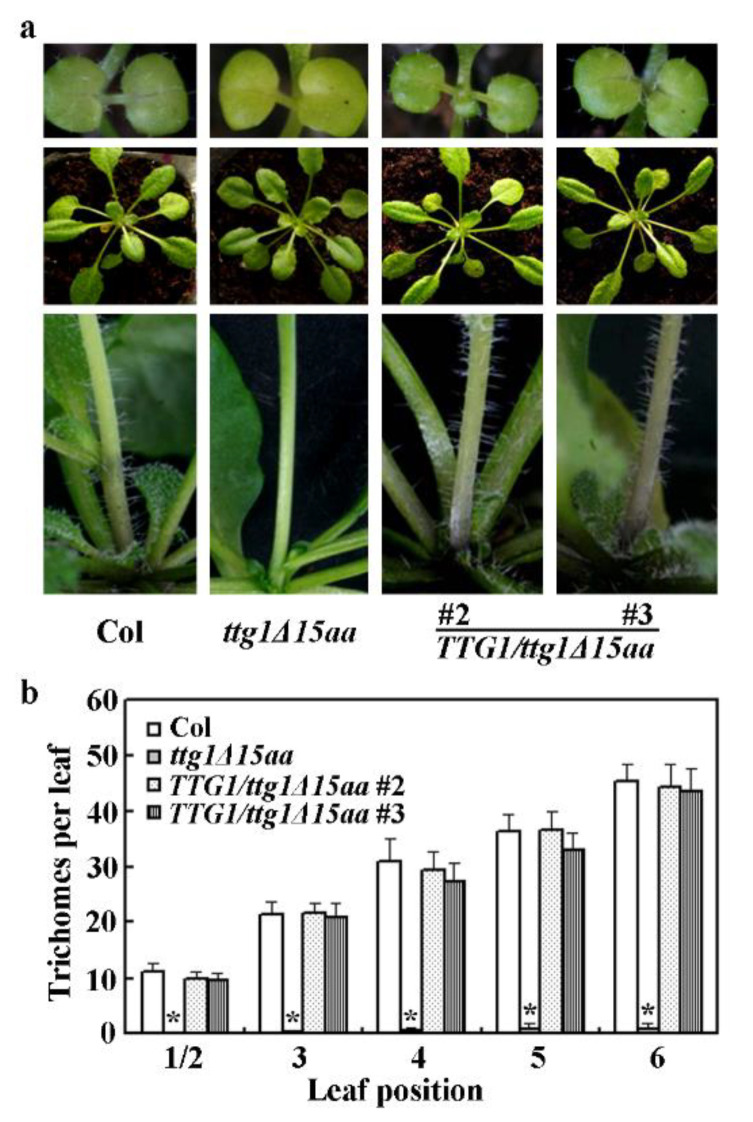
Expression of *TTG1* under the control of its native promoter restores trichome formation in the *ttg1Δ15aa* mutant. (**a**) Trichome formation on leaves of 10-day-old seedlings (up panel), 4-week-old plants (middle panel), and stems of 5-week-old plants (lower panel) of the Col wild type, the *ttg1Δ15aa* mutant, and the *TTG1/ttg1Δ15aa* transgenic plants. Seeds of Col wild type, the *ttg1Δ15aa* mutant, and the *TTG1/ttg1Δ15aa* transgenic plants were germinated and grown in soil pots. Pictures were taken directly or under a Motic K microscope by using an EOS 1100D digital camera at indicated stages. (**b**) Trichome numbers on the first two, third, fourth, fifth and sixth rosette leaves of the Col wild type, the *ttg1Δ15aa* mutant, and the *TTG1/ttg1Δ15aa* transgenic plants. Trichome numbers on the indicated leaves were count under a Motic K microscope. Date represence mean ± SD of 10–14 leaves. *, Significantly different from that in the Col wild type (student’s *t* test, *p* < 0.0001).

To further examine if *ttg1Δ15aa* is a *TTG1* lost-of-function mutant, we performed phenotypic complementation experiment. Transgenic plants expressing *TTG1* under the control of its native promoter were generated in the *ttg1Δ15aa* mutant. We found that trichome formation in the transgenic plants was restored (Figure 3a), and quantitative analysis show that trichome numbers in the transgenic plants examined were restored to a Col wild type level (Figure 3b). In addition, anthocyanin biosynthesis phenotype observed in the *ttg1Δ15aa* mutant was also restored in the transgenic plants (Figure 3a).

**Figure 4:**
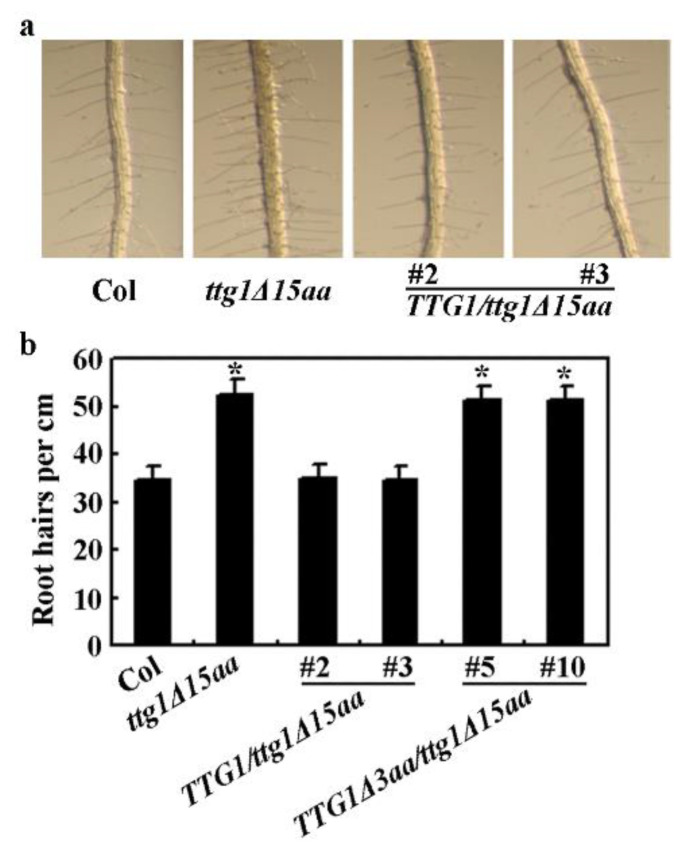
Expression of *TTG1* under the control of its native promoter restores root hair formation in the *ttg1Δ15aa* mutant. (**a**) Root hair formation in the Col wild type, the *ttg1Δ15aa* mutant and the *TTG1*/*ttg1Δ15aa* transgenic plants. Seeds of Col wild type, the *ttg1Δ15aa* mutant, and the *TTG1*/*ttg1Δ15aa* transgenic plants were germinated and grown on ½ MS plates vertically. Pictures were taken from 7-day-old seedlings under a Motic K microscope by using an EOS 1100D digital camera. (**b**) Root hair density in seedlings of the Col wild type, the *ttg1Δ15aa* mutant, the *TTG1*/*ttg1Δ15aa*, and the *TTG1Δ3aa*/*ttg1Δ15aa* transgenic plants. Root numbers were count under a Motic K microscope, and root hair density was calculated. Date represence mean ± SD of 17–18 seedlings. *, Significantly different from that in the Col wuld type (student’s *t* test, *p* < 0.0001).

We also examined root hair formation in the Col wild type, the *ttg1Δ15aa* mutant, and the *TTG1/ttg1Δ15aa* transgenic plants. We found that root hair formation is restored in the *TTG1/ttg1Δ15aa* transgenic plant seedlings (Figure 4a). Quantitative analysis showed that root hair density in the seedlings of the *TTG1/ttg1Δ15aa*, but not the *TTG1Δ3aa/ttg1Δ15aa* transgenic plants, is similar to that of the Col wild type seedlings (Figure 4b). TTG1Δ3aa is a truncated TTG1 protein with a deletion of the last three c-terminal amino acids (see Section 2.5 for details).

**Figure 5:**
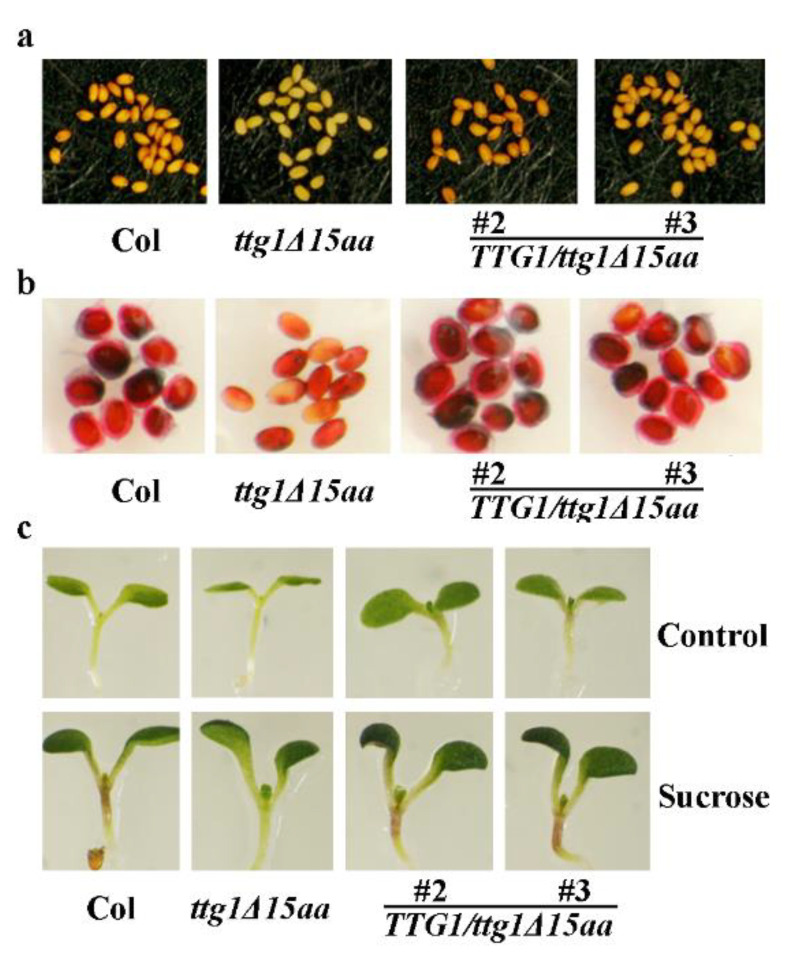
Expression of *TTG1* under the control of its native promoter restores secondary metabolism in the *ttg1Δ15aa* mutant. (**a**) Seed coat color in the Col wild type, the *ttg1Δ15aa* mutant and the *TTG1*/*ttg1Δ15aa* transgenic plants. Pictures were taken under a Motic K microscope by using an EOS 1100D digital camera. (**b**) Mucilage production in the Col wild type, the *ttg1Δ15aa* mutant, and the *TTG1*/*ttg1Δ15aa* transgenic plants. Seeds were stained with 0.01% (*w/v*) Ruthenium red for 2 h, and pictures were taken under a Motic K microscope by using an EOS 1100D digital camera. (**c**) Anthocyanin accumulation in seedlings of the Col wild type, the *ttg1Δ15aa* mutant, and the *TTG1/ttg1Δ15aa* transgenic plants in response to sucrose. Seeds of Col wild type, the *ttg1Δ15aa* mutant, and the *TTG1*/*ttg1Δ15aa* transgenic plants were germinated and grown on half MS plates with or without 3% sucrose. Pictures were taken from 6-day-old seedlings.

We further examined if the metabolism phenotypes in the *TTG1*/*ttg1Δ15aa* transgenic plants including seed proanthocyanidin and mucilage production were recovered in the *TTG1*/*ttg1Δ15aa* transgenic plants. As shown in Figure 5a, the seed color in the transgenic plants is similar to the Col wild type plants, and the seed coat mucilage biosynthesis was also restored in the *TTG1*/*ttg1Δ15aa* transgenic plants (Figure 5b). We also examined anthocyanin biosynthesis in seedlings in response to sucrose. We found that the *ttg1Δ15aa* mutant seedlings failed to produce anthocyanin, whereas the *TTG1*/*ttg1Δ15aa* transgenic plant seedlings accumulated more anthocyanin, similar to that of the Col wild type seedlings (Figure 5c).

### 2.4. TTG1Δ15aa Is Unable to Interact with GL3 and with Regulated Downstream Target Genes

It is well known that TTG1 directly interacts with bHLH transcription factors and together with different R2R3 MYB transcription factors to form MBW activator complexes to regulate downstream gene expression, thereby to regulate different processes including trichome formation, root hair formation, anthocyanin and proanthocyanidin biosynthesis, and seed coat mucilage production [2]. Since the above results suggest that *ttg1Δ15aa* is a *TTG1* loss-of-function mutant, we examined if TTG1Δ15aa may be still able to interact with bHLH transcription factors.

**Figure 6:**
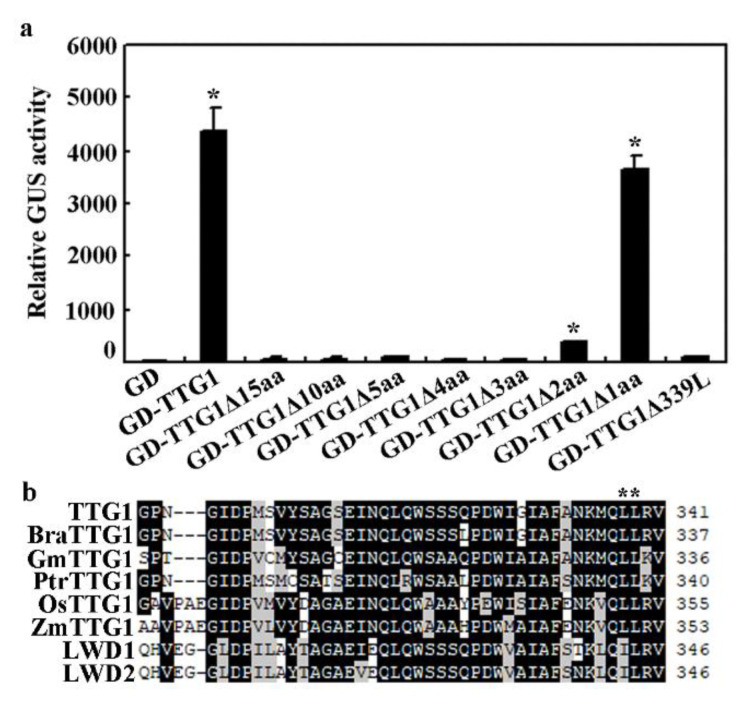
Interaction of full-length and truncated TTG1 with GL3. (**a**) Interaction of TTG1, TTG1Δ15aa, TTG1Δ10aa, TTG1Δ5aa, TTG1Δ4aa, TTG1Δ3aa, TTG1Δ2aa, TTG1Δ1aa, and TTG1Δ339L with GL3 in transfected protoplasts. Protoplasts were isolated from 3 to 4-week-old Col Arabidopsis rosette leaves. Plasmids of the effector genes *GD-TTG1*, *GD-TTG1Δ15aa*, *GD-TTG1Δ10aa*, *GD-TTG1Δ5aa*, *GD-TTG1Δ4aa*, *GD-TTG1Δ3aa*, *GD-TTG1Δ2aa*, *GD-TTG1Δ1aa*, or *GD-TTG1Δ339L* were co-transfected with the reporter gene *Gal4:GUS* and the effector gene *HA-GL3* into protoplasts. The protoplasts were incubated in darkness for 20–22 h, and then GUS activity was assayed. Co-transfection of *GD* with *Gal4:GUS* and *HA-GL3* was used as a control. Data represents the mean ± SD of three repeats. *, Significantly different from that in the GD control (Student’s *t* test, *p* < 0.0001). (**b**) Alignment of the C-terminal amino acids of TTG1, BraTTG1, GmTTG1, PtrTTG1, OsTTG1, ZmTTG1, AtLWD1, and AtLWD2 proteins. Identical amino acids are shaded in black, and similar in grey.

As we have previously shown that the bHLH transcription factor GL3 alone functions as an activator, whereas TTG1 alone is not able to activate reporter gene expression when recruited to the promoter region of the *Gal:GUS* reporter gene by a fused GD domain [40]. Therefore, we used GL3 as an example to examine if TTG1Δ15aa may be still able to interact with bHLH transcription factors. As shown in Figure 6a, the co-transfection of GD-TTG1 and GL3 activated the *Gal4:GUS* reporter gene expression, whereas the co-transfection of GD-TTG1Δ15aa and GL3 failed to do so, suggesting that TTG1Δ15aa is unable to interact with GL3. Assays with truncated TTG1 proteins indicate that the deletion of the last three C-terimnal amino acids or the 339L amino acid is sufficient to abolish the interaction of TTG1 with GL3 (Figure 6a, see Section 2.5 for details). Considering that amino acids in the C-terminus of TTG1 from different plants including Arabidopsis (TTG1), rapeseed (BraTTG1), soybean (GmTTG1), poplar (PtrTTG1), rice (OsTTG1), and corn (ZmTTG1) are highly conserved (Figure 6b), these results suggest that the C-terminus of TTG1 is critical for its functions.

**Figure 7:**
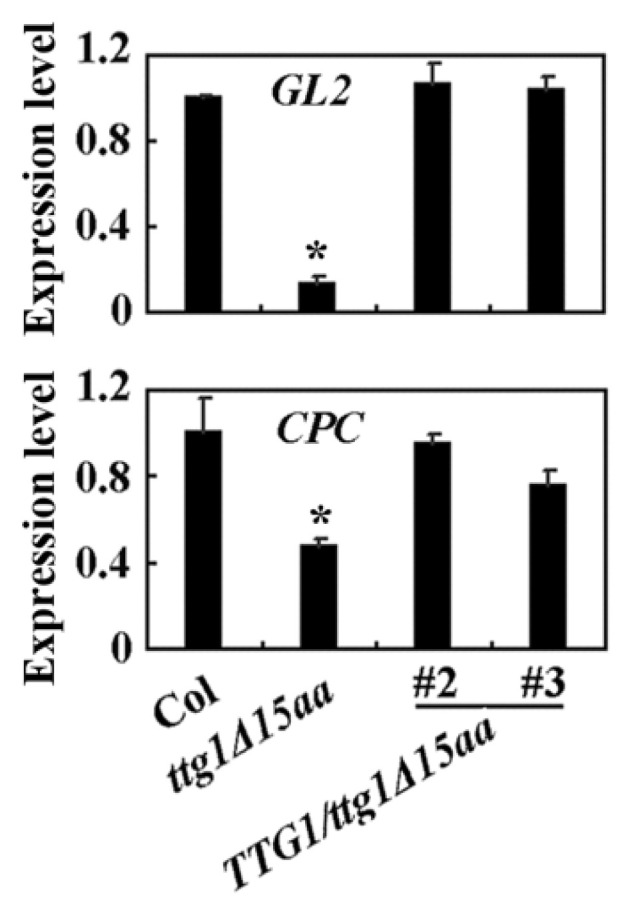
Expression of *GL2* and *CPC* in the Col wild type, the *ttg1Δ15aa* mutant, and the *TTG1*/*ttg1Δ15aa* transgenic plants. RNA was isolated from 10-day-old seedlings and qRT-PCR was used to examine the expression of *GL2* and *CPC*. The expression of *ACT2* was used as an inner control. The expression level of *GL2* or *CPC* in the Col wild type was set as 1. Data represent the mean ± SD of three replicates. *, Significantly different from that in the Col wild type (Student’s *t* test, *p* < 0.01).

We then examined the expression of some TTG1 downstream genes, and we found that the expression of *GL2* and *CPC* was decreased in the *ttg1Δ15aa* mutant, but largely recovered in the *TTG1*/*ttg1Δ15aa* transgenic plants (Figure 7).

### 2.5. Deletion of the Last Three C-Terminalamino Acids Causes Loss-of-Function of TTG1

So far, more than 20 mutants of the *TTG1* gene have been identified, and among them, only a few were caused by premature stop [2], with the *ttg1-1* mutant having the least amino acids deleted at the C-terminus of TTG1 but still showing *TTG1* loss-of-function mutant phenotypes [1,3]. In the *ttg1-1* mutant, the mutation caused a premature stop at the position of the 317Q amino acid, resulting in the production of a truncated TTG1 protein with a deletion of the last 25 C-terminal amino acids [1]. In our case, however, only 15 amino acids were deleted, and it also caused *TTG1* loss-of-function phenotypes in the mutant.

To further examine the minimum amino acids that may cause loss-of-function when deleted, we made truncated TTG1 constructs and examined their interaction with GL3 in transfected protoplasts. We found that the truncated TTG1 proteins with a deletion of the last three or more C-terminal amino acids, including TTG1Δ10aa, TTG1Δ5aa, TTG1Δ4aa, and TTG1Δ3aa, failed to interact with GL3 (Figure 6a). However, TTG1Δ2aa, truncated TTG1 with a deletion of the last two C-terminus amino acids, showed a weak interaction with GL3, whereas the interaction between TTG1Δ1aa, the truncated TTG1 with only the last one C-terminus amino acid deleted, and GL3 is largely unaffected (Figure 6a).

Amino acid alignment of the C-terminus of TTG1 from different plants showed that both of the third and fourth amino acids to the last are L residues and are conserved in all TTG1 proteins, but not LWD1 and LWD2, the two closely related proteins to TTG1 in Arabidopsis (Figure 6b). Previously research has also shown that *ttg1-24*, a mutant with an L339F amino acid substitution in TTG1, produced less trichomes and root hairs and reduced accumulation of proanthocyanidin [42]. In addition, TTG1L339F showed a weak interaction with GL3 [42]. We therefore examined if the deletion of one L amino acid may affect the functions of TTG1 by examining its interaction with GL3. As shown in Figure 6a, GD-TTG1Δ339L failed to interact with GL3 in the transfected protoplasts.

**Figure 8:**
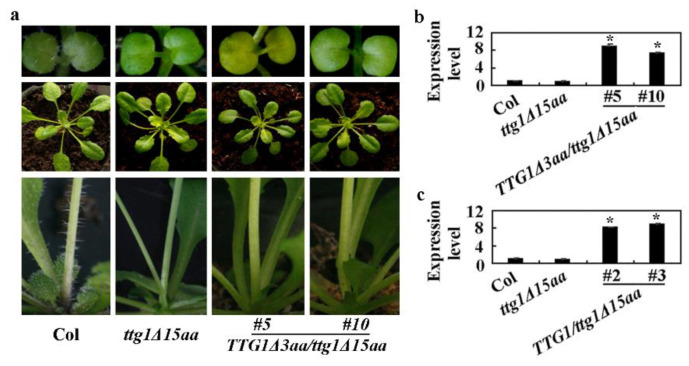
The expression of *TTG1Δ3aa* under the control of *TTG1* native promoter failed to restore trichome formation in the *ttg1Δ15aa* mutant. (**a**) Trichome formation on leaves of 10-day-old seedlings (up panel), 4-week-old plants (middle panel), and stems of 5-week-old plants (lower panel) of the Col wild type, the *ttg1Δ15aa* mutant, and the *TTG1Δ3aa/ttg1Δ15aa* transgenic plants. Seeds of Col wild type, the *ttg1Δ15aa* mutant, and the *TTG1Δ3aa/ttg1Δ15aa* transgenic plants were germinated and grown in soil pots. Pictures were taken directly or under a Motic K microscope by using an EOS 1100D digital camera at indicated stages. (**b**) Expression of *TTG1Δ3aa* in Col wild type, the *ttg1Δ15*aa mutant, and the *TTG1Δ3aa/ttg1Δ15aa* transgenic plants. RNA was isolated from 10-day-old seedlings and qRT-PCR was used to examine the expression of *TTG1Δ3aa*. The expression of *ACT2* was used as an inner control. The expression level of *TTG1Δ3aa* in the Col wild type was set as 1. Data represent the mean ± SD of three replicates. *, Significantly different from that in the Col wild type (Student’s *t* test, *p* < 0.01). (**c**) Expression of *TTG1* in Col wild type, the *ttg1Δ15aa* mutant, and the *TTG1/ttg1Δ15aa* transgenic plants. RNA was isolated from 10-day-old seedlings, and qRT-PCR was used to examine the expression of *TTG1*. The expression of *ACT2* was used as an inner control. The expression level of *TTG1* in the Col wild type was set as 1. Data represent the mean ± SD of three replicates. *, Significantly different from that in the Col wild type (Student’s *t* test, *p* < 0.01).

To further examine if the failure to interact with GL3 may indeed cause the loss-of-function of TTG1, we expressed *TTG1Δ3aa* under the control of *TTG1′*s native promoter in the *ttg1Δ15aa* mutant. We found that the transgenic plants still showed a glabrous phenotype (Figure 8a). The qRT-PCR results show that the expression level of *TTG1Δ3aa* in the *TTG1Δ3aa/ttg1Δ15aa* transgenic plants is elevated (Figure 8b) to a level similar to that of *TTG1* in the *TTG1/ttg1Δ15aa* transgenic plants (Figure 8c).

**Figure 9:**
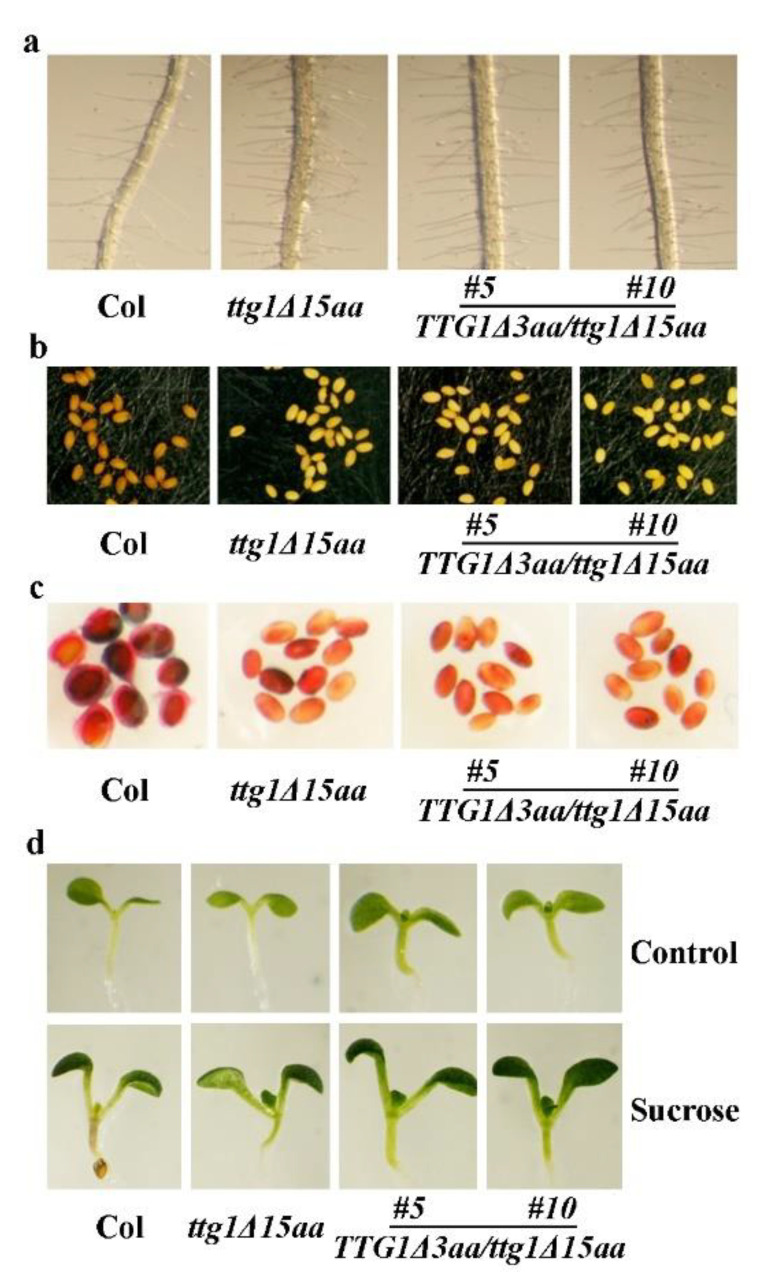
Expression of *TTG1Δ3aa* under the control of *TTG1′s* native promoter failed to restore root hair formation and secondary metabolism in the *ttg1Δ15aa* mutant. (**a**) Root hair formation in the Col wild type, the *ttg1Δ15aa* mutant, and the *TTG1Δ3aa*/*ttg1Δ15aa* transgenic plants. Seeds of Col wild type, the *ttg1Δ15aa* mutant, and the *TTG1Δ3aa*/*ttg1Δ15aa* transgenic plants were germinated and grown on half MS plates vertically. Pictures were taken from 7-day-old seedlings under a Motic K microscope by using an EOS 1100D digital camera. (**b**) Seed coat color in the Col wild type, the *ttg1Δ15aa* mutant, and the *TTG1Δ3aa*/*ttg1Δ15aa* transgenic plants. Pictures were taken under a Motic K microscope by using an EOS 1100D digital camera. (**c**) Mucilage production in the Col wild type, the *ttg1Δ15aa* mutant, and the *TTG1Δ3aa*/*ttg1Δ15aa* transgenic plants. Seeds were stained with 0.01% (*w/v*) Ruthenium red for 2 h, and pictures were taken under a Motic K microscope by using an EOS 1100D digital camera. (**d**) Anthocyanin accumulation in seedlings of the Col wild type, the *ttg1Δ15aa* mutant, and the *TTG1Δ3aa*/*ttg1Δ15aa* transgenic plants in response to sucrose. Seeds of Col wild type, the *ttg1Δ15aa* mutant, and the *TTG1Δ3aa*/*ttg1Δ15aa* transgenic plants were germinated and grown on half MS plates with or without 3% sucrose. Pictures were taken from 6-day-old seedlings.

Root hair formation assays show that the *TTG1Δ3aa/ttg1Δ15aa* transgenic plant seedlings still produced more root hairs (Figure 9a), and a quantitative analysis indicates that the root hair density in the transgenic plant seedlings is similar to that in the *ttg1Δ15aa* mutant seedlings (Figure 4b). Furthermore, the *TTG1Δ3aa/ttg1Δ15aa* transgenic plants still produced yellow-colored seeds, as observed in the *ttg1Δ15aa* mutant (Figure 9b). The ruthenium red staining results show that the seed coats of the *TTG1Δ3aa/ttg1Δ15aa* transgenic plants are still not able to produce mucilage (Figure 9c), and the sucrose treatment suggests that the anthocyanin phenotypes observed in the *ttg1Δ15aa* mutant are also not restored in the *TTG1Δ3aa/ttg1Δ15aa* transgenic plants (Figure 9d).

## 3. Discussion

TTG1 regulates the cell fate determination, flavonoid biosynthesis, and seed coat mucilage production in Arabidopsis via forming MBW activator complexes [2]. Experimental evidence in both yeast and plant cells indicate that TTG1 is able to interact with bHLH transcription factors [39,40]. However, the domain required for the interaction of TTG1 with the bHLH transcription factor remained largely unknown. So far, only the 25 C-terminal amino acids have been shown to affect the interaction of TTG1 with GL3 [39]. We provided evidence in this study that the C-terminus of TTG1 is required for the interaction of TTG1 with GL3 and may play a critical role for its functions in Arabidopsis.

First, we found that *ttg1Δ15aa*, a mutant with a premature stop, which is predicted to result in the production of a truncated TTG1 protein with its last 15 C-terminal amino acids deleted (Figure 2), is a *TTG1* loss-of-function mutant. As the mutant shows a pleiotropic phenotype identical to the CRISPR/Cas9 gene-edited mutant *ttg1-c1* (Figure 1), including glabrous leaves, increased root hairs, transparent testa seed coat, and reduced anthocyanin accumulation and seed coat mucilage production, typical phenotypes observed in the loss-of-function mutants of *TTG1* [1,3,4,5,6]. In addition, the phenotypes observed in the *ttg1Δ15aa* mutant were recovered by the expression of *TTG1* under the control of its native promoter (Figure 3, Figure 4 and Figure 5). Second, TTG1Δ15aa is unable to interact with the bHLH transcription factor GL3 in transfected protoplasts (Figure 6), and the expression levels of TTG1 downstream genes including *GL2* and *CPC* were reduced in the *ttg1Δ15aa* mutant and were largely recovered in the *TTG1/ttg1Δ15aa* transgenic plants (Figure 7). Third, TTG1 with a deletion of the last three C-terminal amino acids or the 339L amino acid alone failed to interact with GL3 (Figure 6). Last but not least, the expression of *TTG1Δ3aa*, which encoded a TTG1 protein with a deletion of the last three C-terminal amino acids, in the *ttg1Δ15aa* mutant failed to recover its phenotypes (Figure 8 and Figure 9). It will be of interest to examine if the deletion of the last few amino acids may affect the binding of TTG1 to its target genes by using ChIP-seq or ChIP-PCR assays.

Even though both TTG1 and R2R3 MYB are able to interact with bHLH transcription factors, thereby forming MBW activator complexes [39,40], only the domains required for R2R3 MYB to interact with bHLH transcription factors have been well studied. The R3 domain has been identified as the interaction domains of R2R3 MYB proteins with bHLH transcription factors, the [D/E]L×2[R/K]×3L×6L×3R in R3 domain has been identified as an amino acid signature for this interaction [39,40,41], and the 92S in the R3 domain has also been found to be required for the interaction of R2R3 MYB transcription factor GL1 and bHLH proteins GL3/EGL3 [43]. As for TTG1, a previous study has only shown that the 25 C-terminal amino acids are required for the interaction of TTG1 with GL3 [39]. Our studies further narrow the region to the last three C-terminal amino acids, and we found that deletion of the 339L amino acid alone fully abolished the interaction of TTG1 with GL3 (Figure 6). Considering that the presence of the WD40 motifs is the only conserved feature of WD40 proteins [15] and WD40 proteins are able to provide a platform for interactions with other proteins [13,14,15], it will be of great interest to examine if the WD40 motifs in TTG1 may be required for its interaction with bHLH transcription factors.

As a matter of fact, a few amino acid substitution mutants of *TTG1* have been found to affect the function of TTG1, including the *ttg1-9* mutant with an S282F substitution [1,44], the *ttg1-11* mutant with a G149R substitution [45], the *ttg1-12* mutant with a G43R substitution [45], the *ttg1-23* mutant with an S197F substitution [42], the *ttg1-24* mutant with an L339F substitution [42], the *urm23* mutant with a G302E substitution [46], and the *ttg1* (*Est*) mutant with an S101F substitution [47]. Among them, amino acid substitution in the *ttg1-9*, *ttg1-11*, *ttg1-23*, and *ttg1* (*Est*) mutant occurred in the WD40 motifs (Figure 2c), and all but *ttg1-23* showed typical *TTG1* loss-of-function mutant phenotypes [1,42,44,45,47], suggesting that WD40 motifs are important to the functions of TTG1. On the other hand, among the three mutants where amino acid substitution occurred outside the WD40 motifs, both *ttg1-24* and *urm23* have amino acid substitutions occurring at the C-terminus and showed weak phenotypes [42,46], but *ttg1-12*, a mutant with amino acid substitution occurred at the N-terminus, also showed typical *TTG1* loss-of-function mutant phenotypes [45]. Examining the interactions of the TTG1 proteins with the above-mentioned amino acid substitutions with bHLH transcription factors may reveal addition domains/motifs required for the interaction of TTG1 and bHLH transcription factors.

## 4. Materials and Methods

### 4.1. Plant Materials and Growth Conditions

The Arabidopsis (*Arabidopsis thaliana*) Columbia-0 (Col) wild type was used for protoplasts isolation, plant transformation, and served as controls for phenotypic analysis. The *ttg1Δ15aa* mutant was isolated from an EMS (ethyl methanesulfonate) mutant pool in the Col wild type background. The *ttg1-c1* mutant was generated by using *CRISPR/Cas9* (*Clustered* *Regularly* *Interspaced* *Short* *Palindromic* *Repeats/CRISPR associated protein 9*) gene editing in the Col wild type plants.

For trichome phenotypic analysis, protoplast isolation and/or plant transformation, seeds of the Col wild type, the *ttg1Δ15aa*, and the *ttg1-c1* mutants, the *TTG1*/*ttg1Δ15aa* and the *TTG1Δ3aa*/*ttg1Δ15aa* transgenic plants were sown into soil pots directly and grew at 22 °C in a growth room with a photoperiod of 16 h light/8 h dark and light density at ~125 µmol m^−2^ s^−1^, as described previously [48].

For RNA isolation, anthocyanin biosynthesis, and/or root hair phenotypic analysis, seeds of the Col wild type, the *ttg1Δ15aa*, and the *ttg1-c1* mutants, the *TTG1*/*ttg1Δ15aa* and the *TTG1Δ3aa*/*ttg1Δ15aa* transgenic plants were bleach sterilized, washed five times with sterilized water, and then plated on plates containing 1% (*w/v*) sucrose and 1/2 Murashige & Skoog (MS) medium solidified with 0.6% (*w/v*) phytoagar (PlantMedia, Dublin, OH, USA). The plates were kept for 2 days in darkness at 4 °C and then transferred to a growth room, as described previously [49].

More than 15 plants/seedlings for each line were used for trichome and root hair phenotype analysis, and the experiments were repeated at least three times with similar results.

### 4.2. RNA Isolation and qRT-PCR

To examine the expression of trichome formation key regulator genes, or expression of *TTG1* or *TTG1Δ15aa*, seedlings of 10-day-old Col wild type, the *ttgΔ15aa*, and the *ttg1-c1* mutants, the *TTG1/ttgΔ15aa* and the *TTG1Δ3aa/ttgΔ15aa* transcription plants were collected, frozen in liquid nitrogen immediately after collection, and kept at −80 °C for RNA isolation.

Total RNA was isolated by using an EasyPure Plant RNA Kit (TransGene Biotech, Beijing, China), and 2 µg total RNA was used to synthesize cDNA by using the EazyScript First-Strand DNA Synthesis Super Mix (TransGene Biotech, Beijing, China) and following the manufacturer’s instructions. Synthesized cDNA was subjected to qRT-PCR analysis of the expression of *TTG1*, *TTG1Δ3aa*, and trichome formation core regulator genes including *GL2* and *CPC*. The expression of *ACTIN2* (*ACT2*) was used as an inner control for qRT-PCR. qRT-PCR was performed on an Applied Biosystems 7500 real time PCR System using SYBR Green/ROX Master Mix (TaKaRa Biomedicals, Dalian, China). Primers used for qRT-PCR examination of the expression of *TTG1Δ3aa* were 5′-CGAGCCAATCTCGGTTCTCA-3′ and 5′-CGAGACGTTTCGGCTCTACA-3′. Other primers used for qRT-PCR have been described previously [49].

### 4.3. Constructs

The reporter construct *Gal4:GUS* and the effector constructs *GD* (*Gal4 DNA Binding Domain*), *GD-TTG1* and the *HA-GL3* used for protoplasts transfection have been described previously [40,50].

To make *GD*-tagged *TTG1Δ15aa*, *TTG1Δ10aa*, *TTG1Δ5aa*, *TTG1Δ4aa*, *TTG1Δ3aa*, *TTG1Δ2aa*, *TTG1Δ1aa*, and *TTGΔ339L* constructs for protoplast transfection, the corresponding ORF sequences were amplified by PCR using *GD-TTG1* plasmid DNA as a template and cloned in frame with an N-terminal GD tag under the control of the double *35S* promoter into *pUC19* vector [50,51]. The forward primer used to amplify all the truncated *TTG1* genes and the reverse primer used to amplify *TTG1Δ3aa* are as described in 4.2, and the reverse primer used to amplify *TTG1Δ15aa* is 5′-CAAGAGCTCCTAATCAGGCTGCGAAGA-3′, for *TTG1Δ10aa* is 5′-CAAGAGCTCCTAAGCAATACCAATCCAATC-3′, for *TTG1Δ5aa* is 5′-CAAGAGCTCCTACATTTTGTTAGCAAAAGCAA-3′, for *TTG1Δ4aa* is 5′-CAAGAGCTCCTACTGCATTTTGTTAG-3′, for *TTG1Δ2aa* is 5′-CAAGAGCTCCTAAAGGAGCTGCATTTTGT-3′, for *TTG1Δ1aa* is 5′-CAAGAGCTCCTATCTAAGGAGCTGCATT-3′, and for *TTGΔ339L* is 5′-CAAGAGCTCTCAAACTCTGAGCTGCATTTT-3′.

To generate *proTTG1:TTG1* and *proTTG1:TTG1Δ3aa* constructs, DNA fragments containing the 1062-bp upstream sequence and the CDS sequence of *TTG1* or *TTG1Δ3aa* were PCR amplified by using DNA isolated from Col wild type seedlings as template and cloned before a *nos* terminator into the binary vector *pPZP211*. The forward primer used for PCR is 5′-CAAGTCGACGGATCAAGATCTTCATATTC-3′, and the reverse primers used for amplifying *TTG1* and *TTG1Δ3aa* are as described in 4.2.

To generate gene editing *CRISPR/Cas9* constructs of *TTG1*, the exon sequence corresponding the CDS sequence of *TTG1* was scanned on CRISPRscan (http://www.crisprscan.org/, accessed on 2 September 2018) to identify potential target sequences, and selected target sequences were evaluated on Cas OFFinder (http://www.rgenome.net/cas-offinder/, accessed on 2 September 2018) to select sequence without potential off-targets. The specific target sequence selected for editing *TTG1* was 5ʹ-GATGTAGAGCCGAAACGTCT(CGG)-3ʹ. The sequence was inserted into the *pHDE* vector to generate *CRISPR/Cas9* construct by following the procedure described previously [52]. The primers used to generate the construct were 5ʹ-GATGTAGAGCCGAAACGTCTGTTTTAGAGCTAGAAATAGCAAGTTA-3ʹ and 5ʹ-AGACGTTTCGGCTCTACATCAATCACTACTTCGACTCTAGC-3ʹ.

### 4.4. Plant Transformation and Transgenic Plants Selection

About 5-week-old Arabidopsis Col wild type or *ttg1Δ15aa* mutant plants with several mature flowers on the main inflorescences were used for *Agrobacterium tumefaciens* *GV3101* mediated plant transformation, and the plants were transformed by using the floral dip method [53].

The Col wild type Arabidopsis plants were transformed to generate *CRISPR/Cas9* edited mutants for *TTG1*. T1 seeds from the transformed plants were collected and plated on plates containing solidified half MS medium with 100 µg/mL Carbenicillin and 30 µg/mL Hygromycin for T1 transgenic plants selection. Gene editing status in T1 transgenic plants identified was examined by amplifying and sequencing the genomic sequence of *TTG1*. T2 seeds collected from gene edited T1 plants were germinated and grew directly into soil pots and used to identify Cas9-free homozygous mutants.

The *ttg1Δ15aa* mutant plants were transformed with *proTTG1:TTG1* and *proTTG1:TTG1Δ3aa* constructs, respectively, for phenotype rescue experiment. T1 seeds from the transformed plants were collected and plated on plates containing solidified half MS medium with 100 μg/mL Carbenicillin and 50 μg/mL Kanamycin for T1 transgenic plants selection. T2 seeds collected from identified T1 transgenic plants were plated on plates containing solidified half MS medium with 25 µg/mL kanamycin to select transgenic lines with 3:1 segregation. T3 seeds collected from individual plants of the lines with 3:1 segregation were plated on plates containing solidified half MS medium with 25 µg/mL kanamycin to select homozygous transgenic plants.

Expression of *TTG1* and *TTG1Δ3aa* in the homozygous transgenic plants was examined by using RT-PCR. Confirmed homozygous overexpression transgenic plants, i.e., *TTG1*/*ttg1Δ15aa* lines #2 and #3, and *TTG1Δ3aa/ttg1Δ15aa* lines #5 and #10, were used for phenotypic analysis.

### 4.5. DNA Isolation and PCR

To examine the mutation occurring in the *ttg1Δ15aa* mutant, leaves of the *ttg1Δ15aa* mutant were collected, and DNA was isolated and used for PCR amplification of *TTG1* genome sequence. PCR product obtained was sequenced, and the sequence obtained was aligned with the wild type *TTG1* sequence.

To examine gene editing status of *TTG1* in the CRISPR/Cas9 gene-edited plants, leaves of T1 transgenic plants were collected, DNA was isolated, and *TTG1* genome sequence was amplified and sequenced. The sequences obtained were aligned with the wild type *TTG1* sequence.

To obtain *Cas9*-free gene edited homozygous mutants, DNA isolated from T2 offspring of confirmed gene edited T1 plants was subjected to PCR amplification of the *Cas9* fragments to identify *Cas9*-free plants, and *TTG1* was amplified and sequenced to identify homozygous mutants. The primers used for amplifying *Cas9* fragment have been described previously [54].

### 4.6. Plasmid DNA Isolation, Protoplast Isolation and Transfection

Plasmid DNA was isolated by using a GoldHiEndoFree Plasmid Maxi Kit (CWBIO, Beijing, China) following the manufacturer’s instructions and used for protoplast transfection.

Protoplasts were isolated from 80–90 rosette leaves collected from 3–4-week-old Col wild type plants and transfected with plasmids of the reporter and effector genes by following the procedure described previously [50].

To examine the possible interaction of TTG1Δ15aa, TTG1Δ10aa, TTG1Δ5aa, TTG1Δ4aa, TTG1Δ3aa, TTG1Δ2aa, TTG1Δ1aa, and TTG1Δ339L with GL3, plasmids of the effector genes *GD-TTG1Δ15aa*, *GD-TTG1Δ10aa*, *GD-TTG1Δ5aa*, *GD-TTG1Δ4aa*, *GD-TTG1Δ3aa*, *GD-TTG1Δ2aa*, *GD-TTG1Δ1aa*, or *GD-TTG1Δ339L* were co-transfected with plasmids of the reporter gene *Gal4:GUS* and the effector gene *GL3* into protoplasts. Co-transfection of *GD* with *Gal4:GUS* and *HA-GL3* were used as a control. The transfected protoplasts were incubated under darkness for 20–22 h at room temperature. GUS activities were measured by using a microplate reader (Synergy™ HT, BioTEK, Winooski, Vermont, USA). Transfection of each combination contains three biological replicates, and the experiments were repeated at least three times with similar results.

### 4.7. Mucilage Production Assays

Seeds of the Col wild type, the *ttg1Δ15aa* and the *ttg1-c1* mutants, and the *TTG1/ttg1Δ15aa* and the *TTG1Δ3aa/ttg1Δ15aa* transgenic plants were stained with 0.01% (*w/v*) Ruthenium red and mounted, as described previously [55]. Mucilage was examined under a Motic K dissecting microscope (MOTIC, Xiamen, China), and pictures were taken by using a digital camera connected to the microscope. At least 30 seeds from each plant were used for the assays, and the experiments were repeated three times with similar results.

### 4.8. Anthocyanin Biosynthesis Assays

Anthocyanin biosynthesis in seedlings of the Col wild type, the *ttg1Δ15aa* mutant, and the *TTG1/ttg1Δ15aa* and *TTG1Δ3aa/ttg1Δ15aa* transgenic plants was assayed by using sucrose treatments, as described previously [43]. At least 30 seedlings from each plant were used for the assays, and the experiments were repeated three times with similar results.

### 4.9. Trichome and Root Hair Formation Assays

For trichome formation assays, seeds of Col wild type, the *ttg1Δ15aa* and the *ttg1-1c* mutants, and the *TTG1/ttg1Δ15aa* and *TTG1Δ3aa/ttg1Δ15aa* transgenic plants were germinated and grown in soil pots. Trichome formation was examined under a Motic K dissecting microscope, trichome numbers on the first pair, third, fourth, fifth, and sixth true leaves were counted, and pictures were taken at indicated growth stages by using a digital camera.

For root hair formation assays, seeds of Col wild type, the *ttg1Δ15aa* and the *ttg1-c1* mutants, and the *TTG1/ttg1Δ15aa* and *TTG1Δ3aa/ttg1Δ15aa* transgenic plants were germinated on plates containing solidified half MS medium and grown vertically. Root hair formation was examined under a Motic K dissecting microscope, numbers of root hairs were counted, and pictures were taken by using a digital camera connected to the microscope.

## 5. Conclusions

Our results in this study indicate that the C-terminus is critical for TTG1’s functions in Arabidopsis, and the deletion of the last three C-terminal amino acids or the 339L amino acid alone is sufficient to abolish the interaction of TTG1 with GL3.

## Data Availability

All data were obtained were presented in this article.
